# Supplementary material for: Synthesis, In Silico Prediction and In Vitro Evaluation of Antitumor Activities of Novel Pyrido[2,3-d]pyrimidine, Xanthine and Lumazine Derivatives
Source: Molecules. 2020 Nov 9;25(21):5205. doi: 10.3390/molecules25215205 (PMC7672615; doi:10.3390/molecules25215205)
Supplement: Supplementary file 1 [file molecules-25-05205-s001.zip › molecules S Figure 7 docking proof.docx]

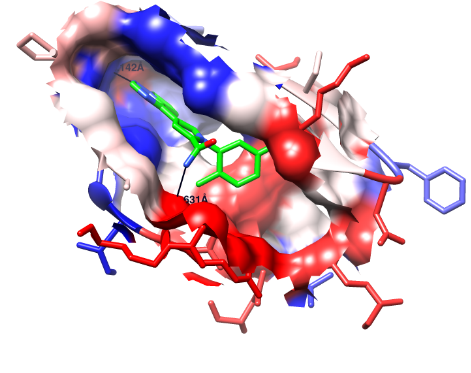

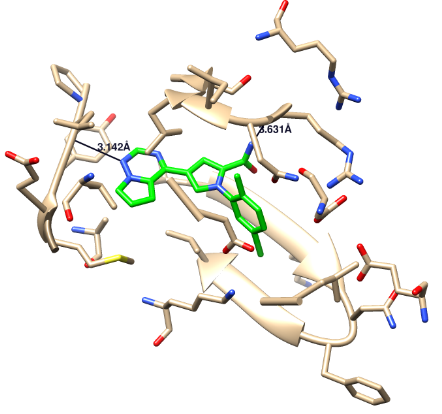

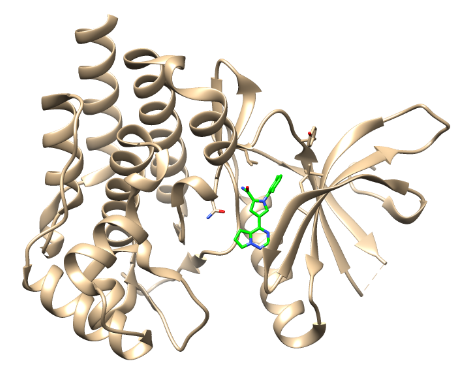


**C**

**A**

**B**

**RL**


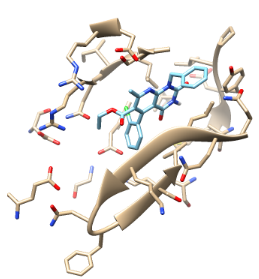


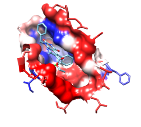

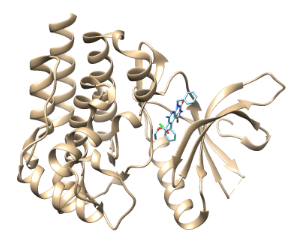


**3b**


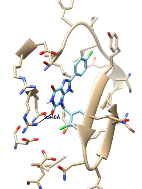

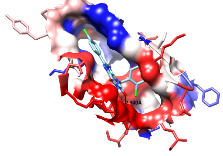


**6c**


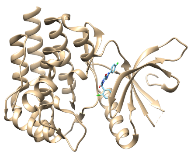


**A**

**B**

**C**

**6d**


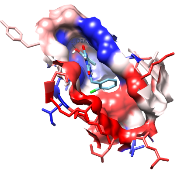

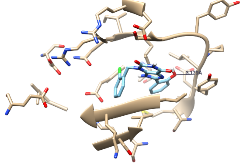

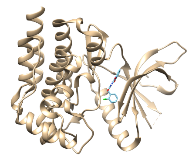


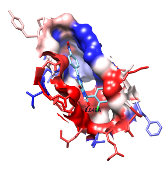

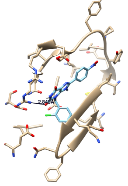

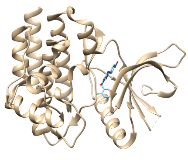


**B**

**A**

**C**

**6e**


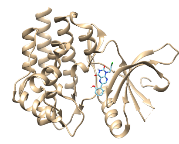

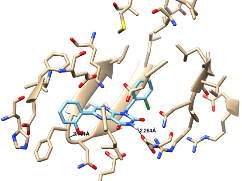

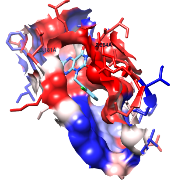


**7c**


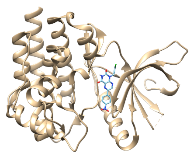

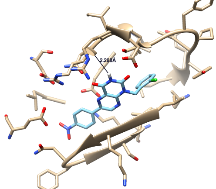

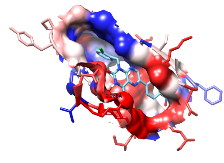


**7d**

**Figure S7:** The interaction of the most promising compounds with Jak2 protein, **A)** 3D interaction, **B)** hydrogen bond formation, and **C)** hydrophobic interaction representation by blue colour.
