# Supplementary material for: Synthesis, In Silico Prediction and In Vitro Evaluation of Antitumor Activities of Novel Pyrido[2,3-d]pyrimidine, Xanthine and Lumazine Derivatives
Source: Molecules. 2020 Nov 9;25(21):5205. doi: 10.3390/molecules25215205 (PMC7672615; doi:10.3390/molecules25215205)
Supplement: Supplementary file 1 [file molecules-25-05205-s001.zip › molecules S. Figure 6 docking proof.docx]

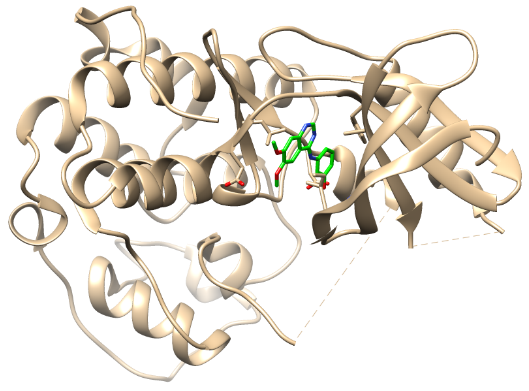

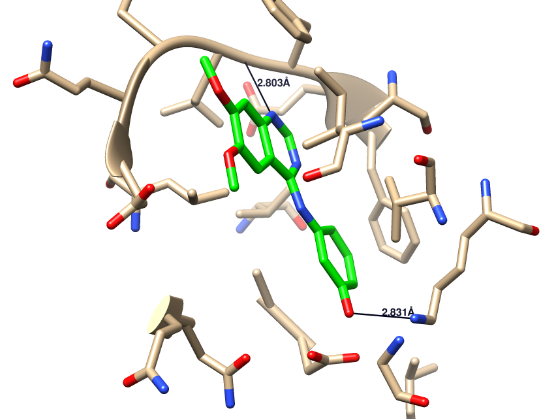

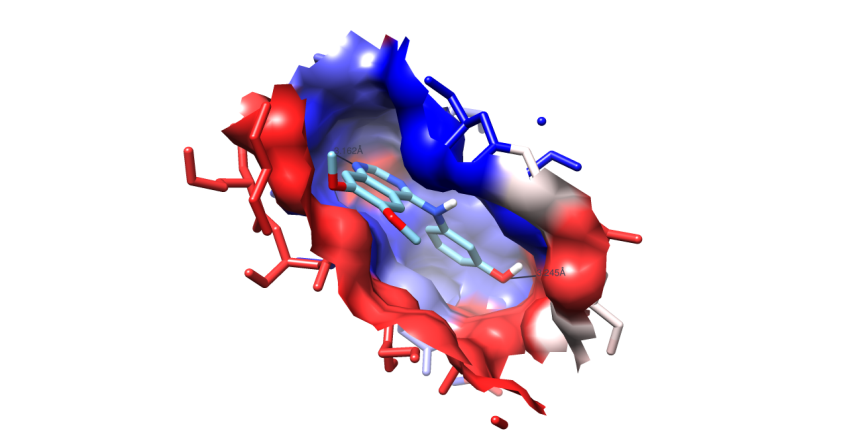

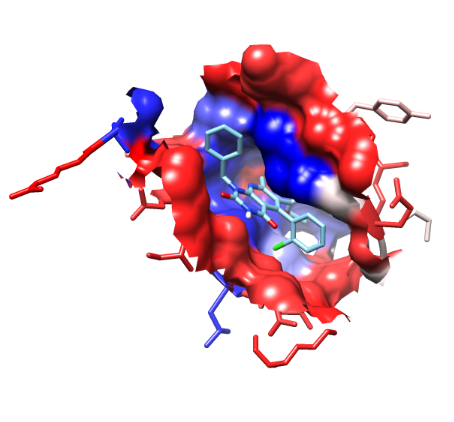

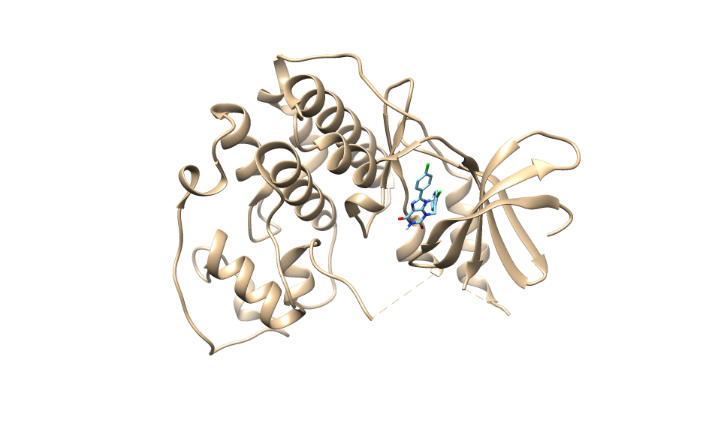

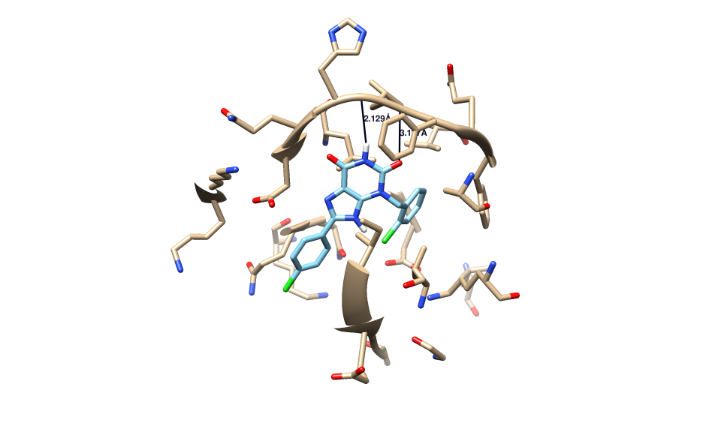

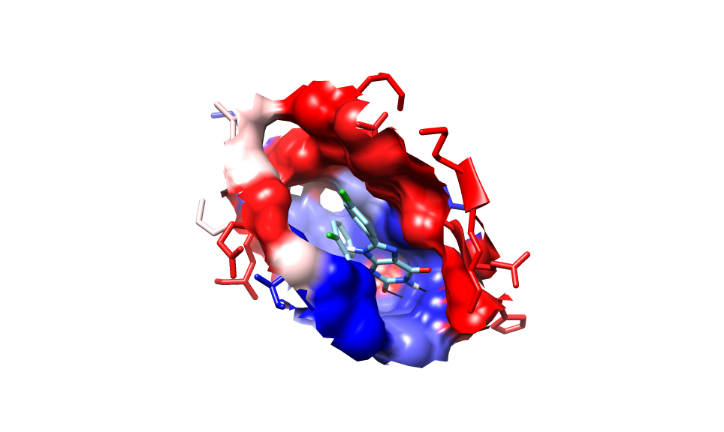

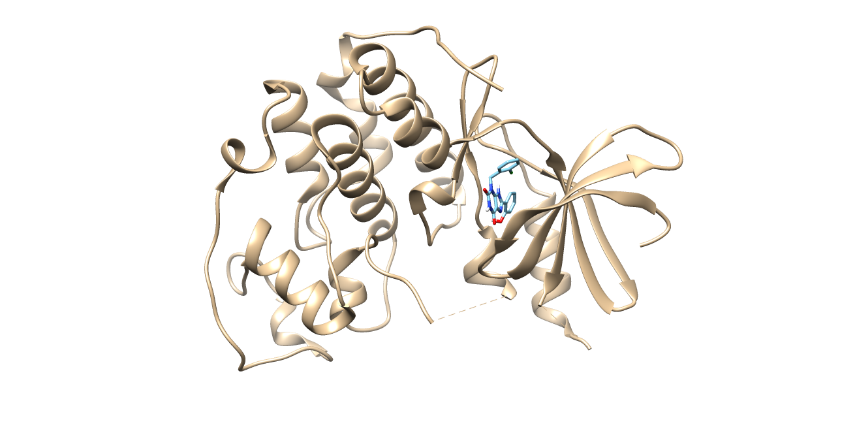

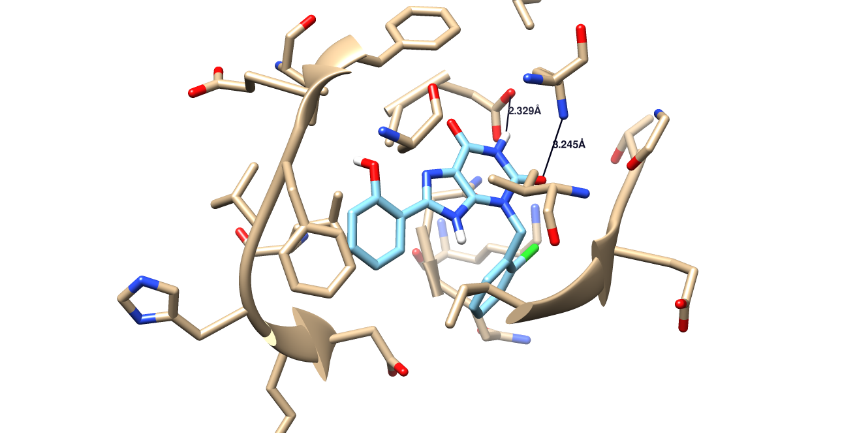

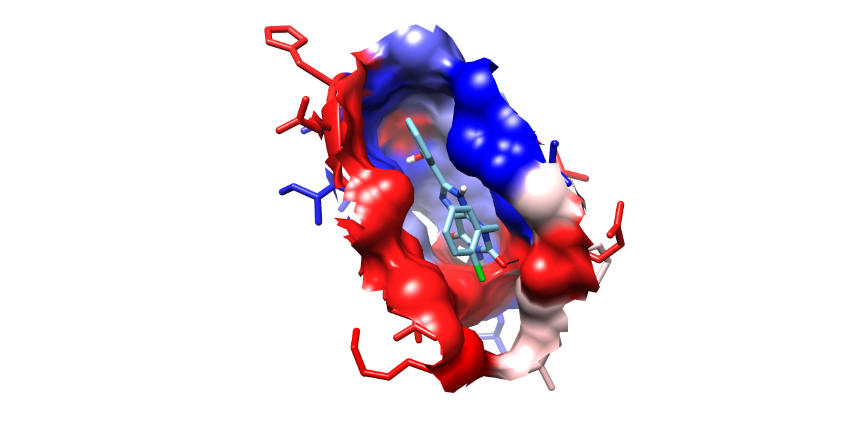

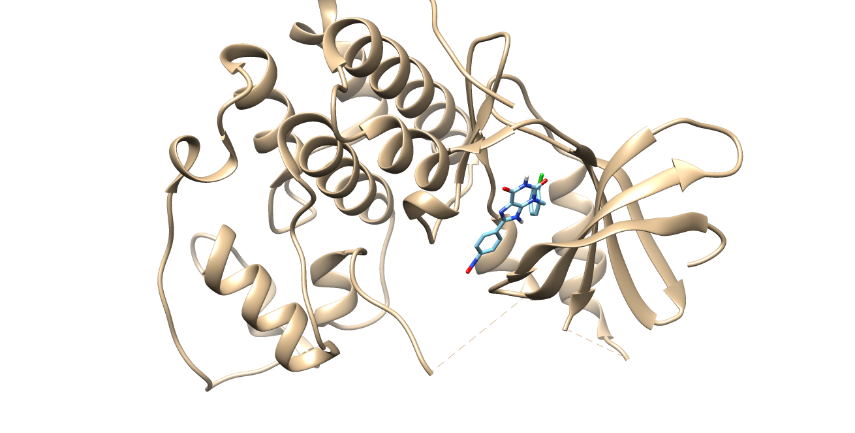

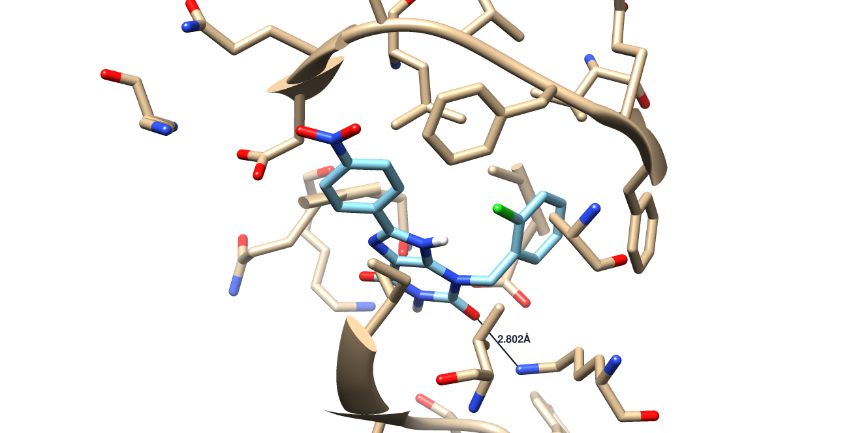

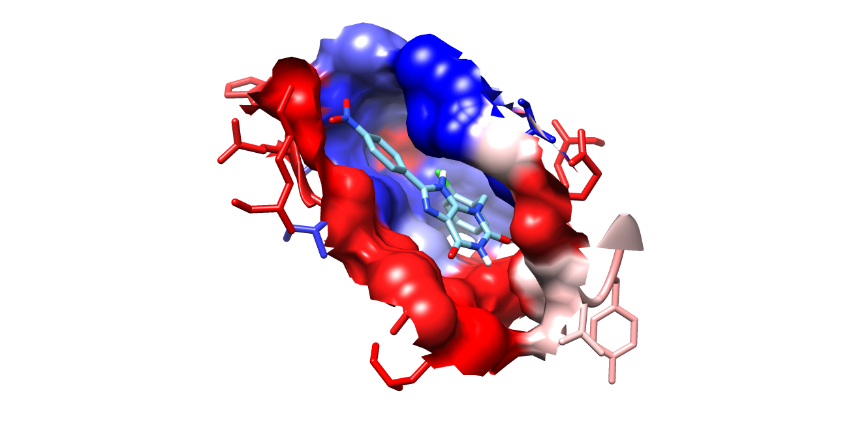


**C**

**B**

**A**

**RL**


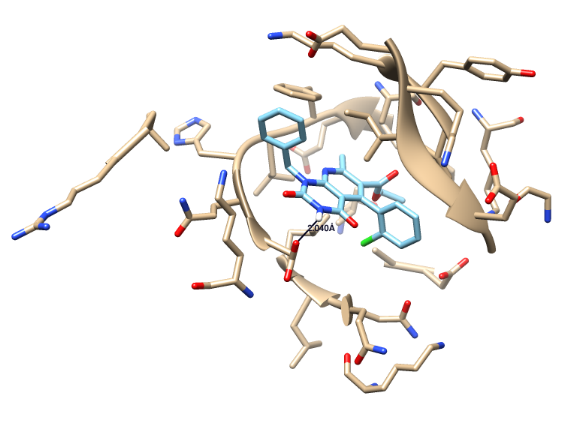

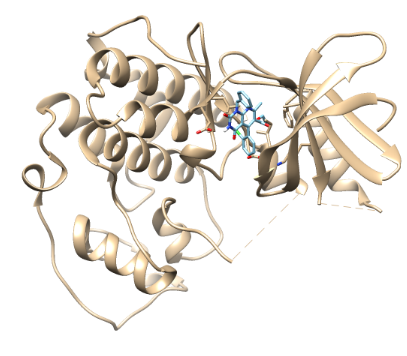


**3b**


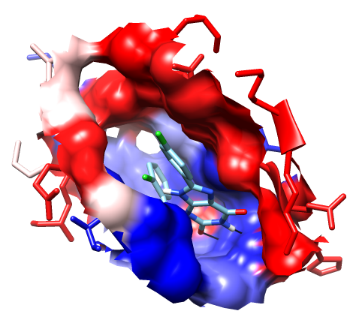

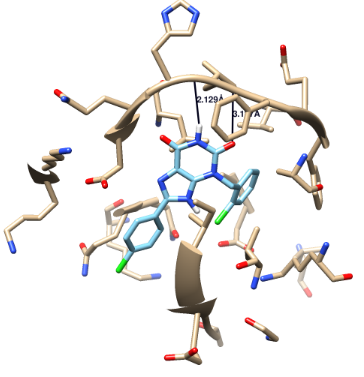

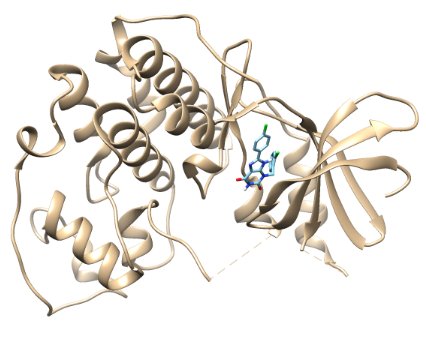


**6c**

**6d**

**6d**

**A**

**B**

**C**


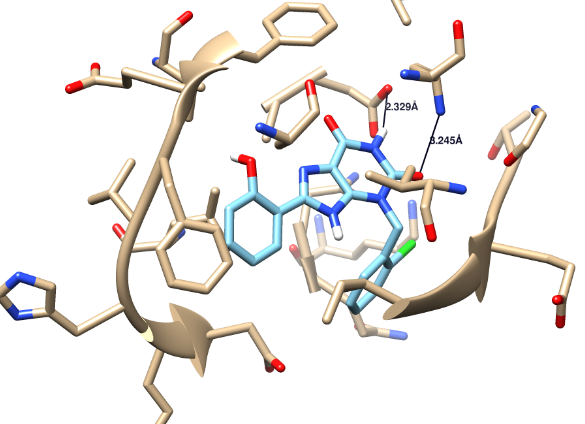

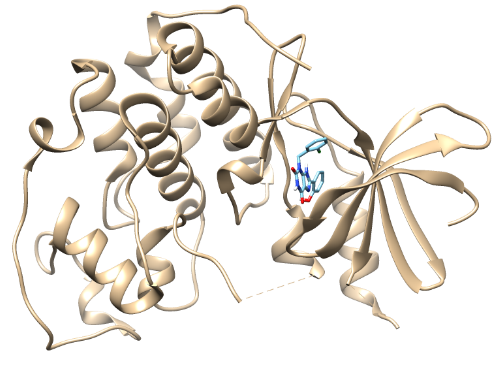


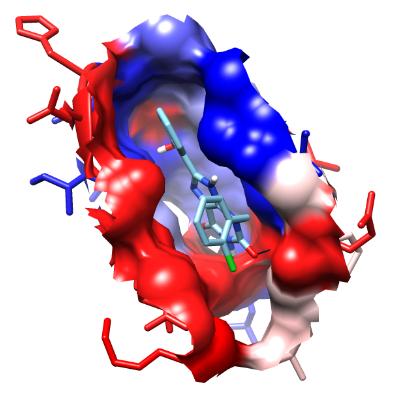


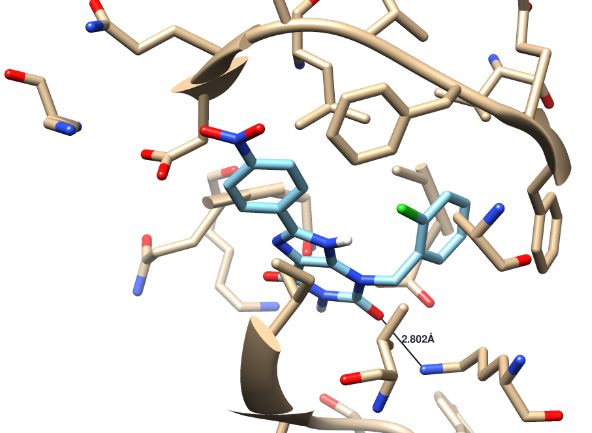


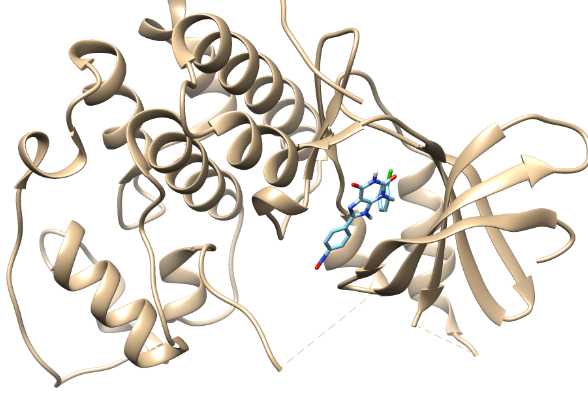

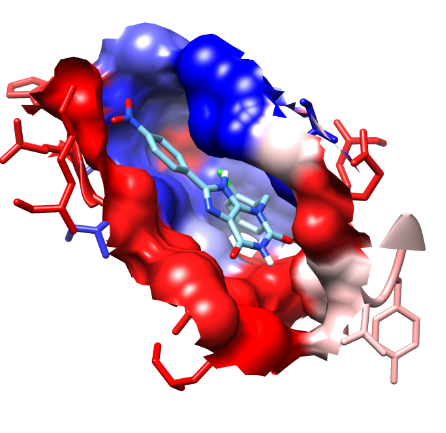


**6e**


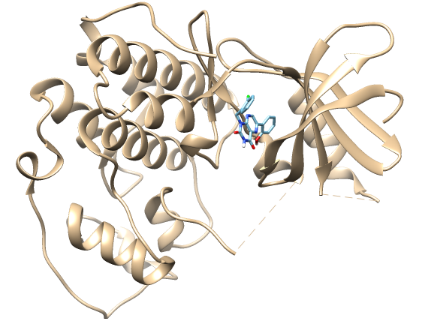

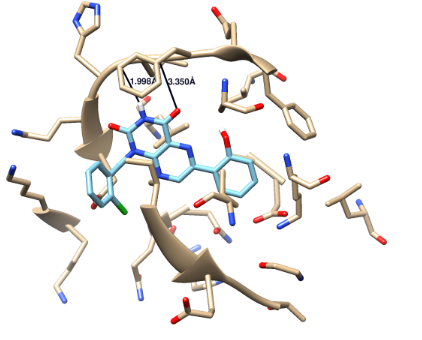

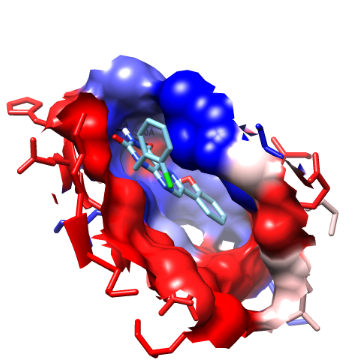

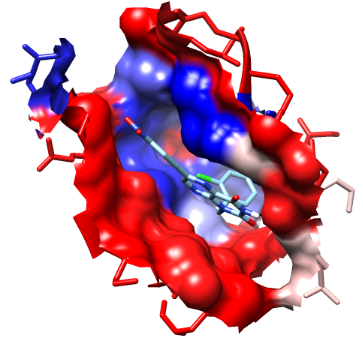


**7c**

**7d**


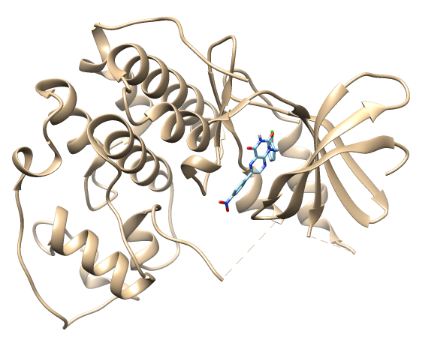


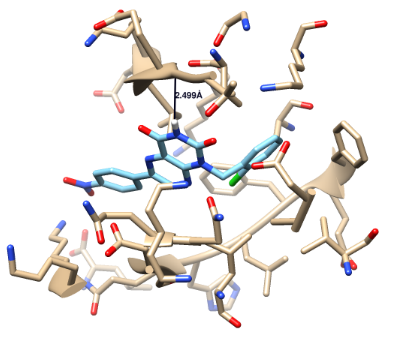


**Figure S6:** The interaction of the most promising compounds with CDK2 protein, **A)** 3D interaction, **B)** hydrogen bond formation, and **C)** hydrophobic interaction representation by blue colour.
