# Supplementary material for: Synthesis, In Silico Prediction and In Vitro Evaluation of Antitumor Activities of Novel Pyrido[2,3-d]pyrimidine, Xanthine and Lumazine Derivatives
Source: Molecules. 2020 Nov 9;25(21):5205. doi: 10.3390/molecules25215205 (PMC7672615; doi:10.3390/molecules25215205)
Supplement: Supplementary file 1 [file molecules-25-05205-s001.zip › Supplementary S1-S5.docx]

**Supplementary file**

**Figure S1:** Free binding of energy values for the tested compounds docked against CDK2 protein binding site.

**Figure S2:** Free binding of energy values for the tested compounds docked against BCL2 protein binding site.

#### Figure S3: Free binding of energy values for the tested compounds docked against Jak2 protein binding site.

**Figure S4:** Free binding of energy values for the tested compounds docked against –MDM2-P53 protein binding site.

**Figure S5:** Free binding of energy values for the tested compounds docked against DHFR protein binding site.
